# The VERB™ Campaign Logic Model: A Tool for Planning and Evaluation

**Published:** 2004-06-15

**Authors:** Marian Huhman, Carrie Heitzler, Faye Wong

**Affiliations:** VERB Campaign, Division of Adolescent and School Health (DASH), National Center for Chronic Disease Prevention and Health Promotion (NCCDPHP), Centers for Disease Control and Prevention; VERB Campaign, Division of Nutrition and Physical Activity, NCCDPHP, CDC, Atlanta, Ga; VERB Campaign, DASH, NCCDPHP, CDC, Atlanta, Ga

## Abstract

The VERB campaign uses a logic model as a tool to share information, to facilitate program planning, and to provide direction for evaluation. Behavior change and communication theories are incorporated to help hypothesize how behavior change might occur. Evaluation of the campaign follows the process of the logic model. The elements of the logic model are described and further explanation “pops up” as the reader rolls over the graphic of the logic model.

## Introduction

A logic model is a picture of how planners think their program is going to work. It is a systematic, visual way to present the elements of a project and its desired outcomes. Also called a program’s “theory of action,” the logic model expresses not only the obvious components, such as the program’s activities, but also the underlying assumptions and theoretical framework of the program’s interventions (1). Throughout the life of VERB™ (2), the logic model has been a tool for campaign planners to communicate with stakeholders, participating creative agencies, and evaluators. As a road map that guides campaign planning, message development, and outcome evaluation, the logic model has changed as the strategy of VERB has evolved in response to research and results from VERB’s evaluation. Here we describe the elements of the VERB logic model, summarize key theories that have influenced its development, and link evaluation activities to the components of the model. The reader can scroll through the graphic of the logic model for additional information on the model’s components.

## Elements of the Model

**Figure:**
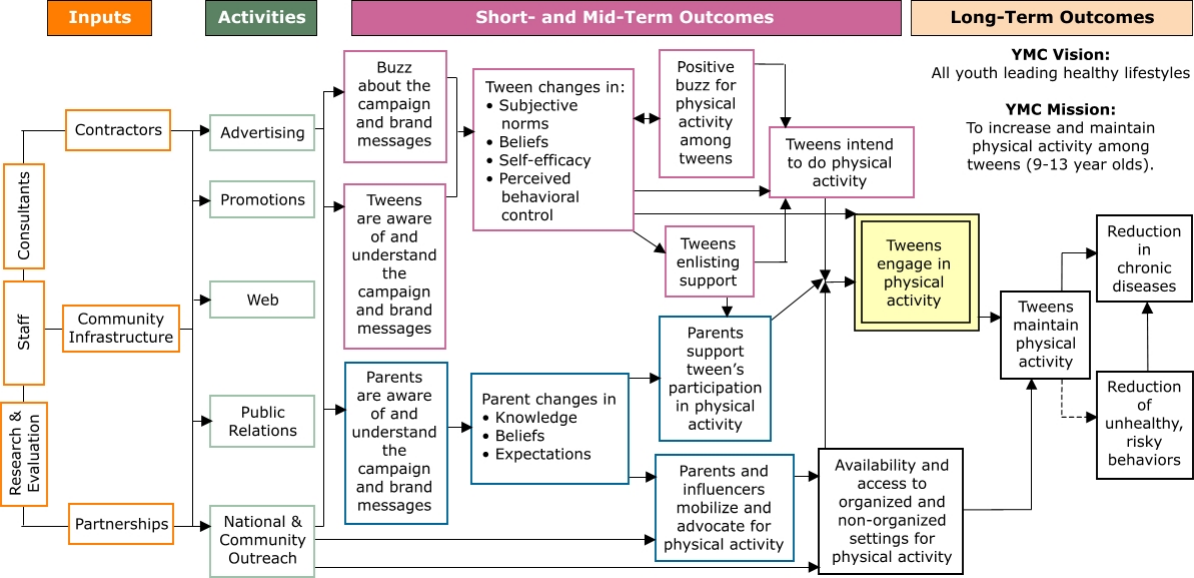
Enlarged image and descriptive text VERB Campaign Vision: All youth leading healthy lifestyles. VERB Campaign Mission: To increase and maintain physical activity among tweens (children aged nine to 13 years). The Inputs of the campaign are:
ConsultantsStaffResearch & EvaluationContractorsCommunity InfrastructurePartnerships All Inputs contribute to campaign activities. Campaign activities include:
AdvertisingPromotionsWebPublic RelationsNational & Community Outreach All Activities lead to short-term outcomes for both tween and parent audiences. The short-term outcome for the campaign is tween and parent awareness of and buzz about the campaign brand and its messages. Awareness and buzz lead to mid-term outcomes, which include changes in:
Subjective normsBeliefsSelf-efficacyPerceived behavioral control The logic indicates that if these changes occur, positive buzz will be created among tweens about physical activity. Tweens will enlist support from their parents and generate intentions to take part in physical activity. Awareness and understanding of the campaign and brand messages by parents leads to changes for parents in Knowledge, Beliefs, and Expectations. The logic indicates that if these changes take place among parents, and if tweens are enlisting their parents' support, parents will support tweens' participation in physical activity, and Parents and influencers will mobilize and advocate for physical activity. The mobilization of parents and influencers and advocacy for physical activity as well as national and community outreach lead to the Availability and access to organized and non-organized settings for physical activity. Tweens' behavioral intention as well as parent support and available and accessible settings are likely to result in tweens engaging in physical activity. The Long-Term Outcomes include tweens engaging in and maintaining physical activity, thereby reducing chronic diseases. The model also indicates a possible displacement strategy: tweens who participate in physical activity may also have a Reduction of unhealthy, risky behavior.

When mapping out an activity, it is often best to start with the desired destination. For VERB, the destination is expressed in the model under the heading of *Long-Term Outcomes* by the statement of broad vision, *All youth leading healthy lifestyles*, and specific mission, *To increase and maintain physical activity among tweens* (children aged nine to 13 years). The Congressional language that appropriated funding for VERB included the words of the vision statement. Campaign planners selected physical activity and the tween age group as VERB’s focus to reach the vision. The endpoint of the logic model, the box titled *Reduction in chronic diseases*, reflects the desired long-term impact of VERB’s vision, an outcome that is broader than the scope of VERB and that will take years to realize. Nonetheless, the goal of tweens engaging in and maintaining physical activity is within reach of the planned five years of the VERB campaign and will affect the development of chronic diseases as children age into adulthood (3).

### Inputs

The logic model reads from left to right, beginning with *Inputs*, or fundamental resources, to the campaign. These inputs include *Consultants* (experts in marketing, youth physical activity, and evaluation), organizational resources such as project *Staff*, and the *Research & Evaluation* infrastructure at the Centers for Disease Control and Prevention (CDC). *Contractors* represent a major input and include general-market creative and public relations agencies, creative agencies specializing in ethnic markets, and an evaluation contractor. *Community Infrastructure* is an input that expresses the importance of context for the child’s engagement in physical activity. *Partnerships* provide an essential resource for guiding, supporting, and extending the reach of the VERB campaign.

### Activities

*Activities* are the products that result from the project’s inputs. As a media campaign, VERB focuses on both paid and donated *Advertising* in the form of television and radio commercials, print advertisements, and out-of-home ads (e.g., billboards, mall kiosks). Important *Promotions* include special events, in-school promotions, contests, and sweepstakes. VERB hosts three Web sites, one each for tweens, parents, and partners. Additionally, VERB provides and monitors information through *Public Relations* activities. *National & Community Outreach* has been actualized by engaging partners such as the YMCA and Boys & Girls Clubs and by mobilizing community-based constituents whose goals are aligned with those of VERB.

### Short-term and mid-term outcomes

#### Tweens

*Activities* lead to *Short-Term Outcomes* and then to *Mid-Term Outcomes* for both the tween and parent audiences. The first boxes, sometimes called proximal outcomes, relate to tween and parent awareness of the campaign brand and its messages. An underlying assumption of the VERB model is that individuals must first attain a high degree of awareness to achieve behavior change. Awareness for VERB begins with the VERB brand, the vehicle for message delivery. If a brand is created that is “cool” to the target, tweens will talk — or generate *Buzz about the campaign and brand messages* — further heightening interest in the brand and the messages.

VERB’s messages appeal to tweens’ needs to have fun, to enjoy themselves while being active with friends, and to have confidence that they can be physically active. These messages are linked to desired changes in how tweens experience physical activity in their lives, depicted in the next box of the logic model. Specifically, VERB aims to change tweens’ a) *Subjective norms* (the belief that important referents approve or disapprove of a behavior); b) *Beliefs*, especially in the benefits of physical activity; c) *Self-efficacy*, or confidence about being physically active; and d) *Perceived behavioral control*, or how strongly tweens believe they can engage in physical activity even if there are barriers to overcome.

Proceeding to *Mid-Term Outcomes* for tweens, VERB planners believe that as tweens internalize the benefits of physical activity, they will act on these beliefs by a) generating *Positive buzz for physical activity among tweens* (now about physical activity, not just the VERB ads); b) *enlisting support* of parents to help them be physically active; c) going through a phase of *intend[ing] to do physical activity*; or d) going immediately to a long-term outcome of *engage[ment] in physical activity*. Some children, especially younger children, may skip c) (intention) and go directly to engagement in physical activity because they are not deliberate in their decision-making process, or parents may sometimes intervene prior to intention.

#### Parents

For parents, short- and mid-term outcomes also begin with message awareness. VERB messaging for parents is meant to work differently, however, than it does for tweens. Because VERB must be kept genuine as a cool “by kids, for kids” brand, the brand is de-emphasized for parents. Parent-directed messages aim to affect how parents prioritize physical activity in their child’s life, giving the parents specific recommendations for physical activity for their child, helping them develop skills on how to verbally and nonverbally support their child’s physical activity, urging them to expect their child to be physically active, and, finally, encouraging parents to be physically active with their child. Campaign planners hypothesize that as parents internalize changes in *Knowledge, Beliefs*, and *Expectations*, parents will support *tween’s participation in physical activity*, enhanced by tweens *enlisting* support from them. As depicted in the model, planners also expect that as parents prioritize their child’s physical activity needs, the parents, as well as other influencers of tweens (e.g., coaches, teachers), will *mobilize and advocate for physical activity*.

VERB views parents and other influencers as important forces in helping to ensure that options and opportunities are available for children to be active. For children to engage in organized or free-time activities, they need teams, clubs, and safe and appealing places to be active. As depicted in the model, VERB perceives *Activities* of *National & Community Outreach* as essential to improving *Availability and access to organized and non-organized settings for physical activity*. VERB acknowledges that places to be active are part of the critical socioecological perspective that surrounds the campaign and affects its immediate and long-term success.

### Long-term outcomes

The box *Tweens engage in physical activity* is framed by a double line to indicate that it is the primary distal outcome of the VERB campaign. This box represents a broad conceptualization of engagement in physical activity that ranges from inactive tweens doing at least something to minimally to moderately active tweens doing more; it also includes tweens trying a new activity. VERB ads seek to motivate the minimally active tween and to keep tweens in general interested in physical activity. Promoting new activities may be an important route for some tweens to experience the benefits of physical activity. As depicted in the logic model, intention may precede engagement; parent support and availability and access to physical activity settings are important in facilitating tweens’ engagement in physical activity.

Tweens being physically active is a necessary but not sufficient outcome of the VERB campaign. Thus, the double-lined box leads to the endpoint of the VERB campaign, the critical and sustained effect of VERB on tweens: *Tweens maintain physical activity*. VERB planners believe that for tweens to maintain physical activity, communities must sustain efforts to provide safe and appealing settings for tweens to be physically active, depicted by the arrow connecting the box *Availability and access to organized and non-organized settings for physical activities* to the box *Tweens maintain physical activity*.

## Theoretical Constructs

Theories related to social marketing and behavioral change have played an important role in developing the logic model. Research in marketing, communication, and physical activity provided a theoretical structure for advising the creative agencies on developing the VERB messages and for helping evaluators hypothesize a model of change. The logic model’s boxes and directional arrows represent the underlying assumptions and the beliefs or theories about how children will change in response to VERB advertising. Below, we describe the theoretical framework that drives the VERB logic model.

### Branding theory

Branding theory (4) posits that the target audience will develop a relationship with the brand that 1) begins with association with brand attributes, 2) builds affinity to those attributes over time, and 3) results ultimately in long-term loyalty to the brand. Hypothesized steps in building the VERB brand relationship include the following:

- Association with an image that is cool, fun, and socially appealing.
- Production of a positive affective response, such as “I like VERB” or “I want to be VERB,” and a positive cognitive response, such as “I think I can do [what is seen in the advertisement], too.”
- Integration of these associations into the child’s identity: for example, “I am a cool, fun kid because I am a VERB kid.”
- Evolution of the child’s beliefs — with repeated exposure — toward, for example, “I want to do X and I will try X because I am a cool, fun VERB kid.”
- Motivation to change through the VERB brand occurs because VERB supports the child’s existing motives, needs, aspirations, and values. The effect would be that the child first juxtaposes VERB values with his or her own, and then shifts toward adopting the VERB motives, needs, aspirations, and values.

Support for these processes is provided by researchers with the *truth*™ anti-smoking campaign, who have posited that through internalization of the “*truth* brand,” youth adopt self-images as a “*truth* teen” that lead to positive dispositions not to smoke (5). Similarly, VERB assumes that children will adopt behaviors portrayed in the ads that support a self-image of being physically active through a process known as “identification” — how much children want to be like the people they see on television. Identification is associated with important antecedents to behavior change, such as expectations of benefits, and is especially salient with younger children (6).

### Message design

Theories of message design help explain the steps for building awareness of a brand like VERB. For example, if branding evokes a high level of interest and identification with the advertisement, then information processing theory, such as the Elaboration Likelihood Model (7), suggests that the child would be willing to exert more cognitive effort to pay attention to the ad, understand it, and actively process its message. Messages provoke active processing when the presentation of content is unusual, unfamiliar, or novel (8) — all key features of the VERB advertisements — and when there is a discrepancy between expectation and reality, such as with the “Paint the Town” VERB promotions in which a water tower is wrapped to look like a soccer ball.

VERB campaign planners decided early on that VERB would be a positive campaign with advertising exclusively characterized by laughter, play, fun, and enjoyment of physical activity. Message designers believe that creating positive feelings in the child will lead to immediate, but also enduring, influence over later cognitive processing (9). Positive appeals are good at attracting attention; they invoke “approach” behaviors, meaning they make it more likely that the audience will be receptive to the forthcoming message. Monahan has asserted that positive appeals are more likely to be recalled, to change attitudes, and to lead to compliance with the desired behavior (9). Moreover, media campaigns that promote commencement of a desirable, positive behavior show larger effect sizes than cessation campaigns that attempt to extinguish a risky or undesirable behavior (10).

Articulating how VERB would lead to change in physical activity behaviors was especially challenging because most health-related behavior change theories are still being tested for their applicability to children. Two theories, the theory of planned behavior (11) and social cognitive theory (12), have been used to plan or test interventions in children and were applied to VERB. The logic model incorporates elements of the two theories into boxes under *Short- and Mid-Term Outcomes*.

### Theory of planned behavior

The theory of planned behavior proposes that one can predict people’s intention to perform a behavior from the attitudes they hold toward that behavior, from a measure of their subjective norms, and from the control they have to perform the behavior (13). The theory proposes that intentions correlate with observed actions (14). A study of Canadian youth reports support for the association of norms, attitudes, and behavioral control with intentions to be physically active (11).

### Social cognitive theory

While the theory of planned behavior is a personal-level theory, social cognitive theory emphasizes the interplay of intrapersonal factors, environment, and behavior (15,16). Intrapersonal factors have cognitive, affective, and biological aspects. Environmental factors encompass the immediate social environment, such as teachers, peers, and parents, in addition to physical environments, such as the presence and appeal of a backyard, driveway, or neighborhood. Principles of social cognitive theory that are especially applicable to child physical activity behaviors include 1) perceptions of the environment, knowledge, and skill to perform an activity; 2) others’ observations of and reinforcement for activity trial; 3) beliefs about the likely outcomes of a behavior; and 4) the values that the child places on the outcome. In addition, self-efficacy (the confidence of the child to perform the behavior) is a critical part of the theory and has been shown to be an important prerequisite for the physical activity of children (17).

### Information processing theory

Another theory that guided campaign strategists to manage expectations for the size of behavioral effects is McGuire’s hierarchical steps of information processing (18). McGuire’s model posits that the impact of persuasive communication is mediated by three broad stages of message processing: attention, comprehension, and acceptance. Attention depends on exposure and awareness; comprehension is predicated on understanding the message; and acceptance includes intention and, finally, behavior change. In McGuire’s model, because of the inherent variability in how people process media messages, a percentage of the audience is lost at each stage. Thus, high levels of exposure and awareness are needed to create measurable population effects.

## Evaluation

The logic model has been an important tool in bringing rigor and direction to VERB’s process and outcome evaluations. In the beginning stages of the campaign, the exercise of mapping how the program would work brought clearer vision and purpose to campaign efforts, which in turn eased the development of measurable objectives, making evaluation more efficient and productive. Every box of the logic model beyond the *Inputs* section has been evaluated for process or outcome. For example, one of VERB’s major promotional events was evaluated with on-site intercept interviews and a follow-up survey. In addition, VERB’s creative agencies use the VERB Brand Tracking Survey primarily to assess key features of the brand’s resonance with tweens. VERB’s staff use the tracking survey continuously to track the numbers of tweens who have an awareness and understanding of VERB and its messages. They also use the survey to track multiple qualitative features like “wear-out” of commercials and to help modify the channels of VERB’s messages.

The instruments that measure campaign outcomes reflect the constructs expressed in the *Short- and Mid-Term Outcomes* boxes. The primary outcome tool, the Youth Media Campaign Longitudinal Survey includes items that tap tweens’ perceptions of social norms, self-efficacy, beliefs, and perceived behavior control. Survey items measure tweens’ intentions to be active and perceptions of parental support as well as parental knowledge, attitudes, and behavior. The survey also includes five measures of physical activity behavior to evaluate the long-term outcomes of the campaign. (The survey as well as other evaluation resources are available at http://www.cdc.gov/youthcampaign/research/index.htm.)

Finally, a conceptual and practical benefit of a logic model is reinforcement of the idea among VERB program staff that projects like VERB represent a continuous process where results of evaluation are used to modify *Inputs* and *Activities*. To maximize impact of the VERB campaign on the physical activity levels of American tweens, VERB planners are continuously engaged in this feedback loop, responding to audience research data, the monthly tracking study, and the outcome evaluation to refine VERB messages and their delivery.

## Summary

The VERB campaign’s logic model is a learning and management tool that began long before the VERB brand existed. The logic model is a workhorse for the VERB campaign; it serves as an effective tool for sharing knowledge among stakeholders, creative agencies, and program staff, contributes to more effective programming, and provides conceptual structure and practical direction for VERB’s evaluation.
